# Treading the unusual path: a rare case of fistula-in-ano extending up to thigh

**DOI:** 10.1093/jscr/rjaf172

**Published:** 2025-04-03

**Authors:** Amol A Gupta, Alok M Gupta, Swati Deshpande, Aditya Sriharsha Pedaprolu, Tushar O Dahmiwal, Jhanwi S Khurana

**Affiliations:** Datta Meghe Institute of Higher Education and Research Deemed to be University, General Surgery, Sawangi Meghe, Wardha 442001, Maharashtra, India; Rashtriya Ayurveda Vidyapeeth, Sharsutra / Surgery, Dhanvantari Bhavan, Road No 66, Punjab Bagh, New Delhi 110026, India; Datta Meghe Institute of Higher Education and Research Deemed to be University, General Surgery, Sawangi Meghe, Wardha 442001, Maharashtra, India; Datta Meghe Institute of Higher Education and Research Deemed to be University, General Surgery, Sawangi Meghe, Wardha 442001, Maharashtra, India; Datta Meghe Institute of Higher Education and Research Deemed to be University, General Surgery, Sawangi Meghe, Wardha 442001, Maharashtra, India; Datta Meghe Institute of Higher Education and Research Deemed to be University, General Surgery, Sawangi Meghe, Wardha 442001, Maharashtra, India

**Keywords:** complex fistula, fistula-in-ano, fistulectomy

## Abstract

The perianal region is typically the site of a fistula-in-ano tract’s opening, though this is not always the case. Complex fistulas are therefore very difficult to treat and frequently the cause of recurrence. This article describes a unique fistula-in-ano that extended posteriorly to the mid-thigh and was successfully treated with multiple modalities and secondary healing.

## Introduction

An anal fistula is a chronic tract between the skin around the anus and the rectum [1]. It can be associated with specific illnesses such as rectal duplications, tuberculosis, cancer, and Crohn’s disease [2]. However, most cases are considered non-specific and idiopathic [3]. Diagnosing and treating a complicated fistula-in-ano is challenging due to the increased risk of recurrences. In this case, we present the multimodal management of a difficult perianal fistula extending to the thigh using a diode laser and vessel sealer in procedures such as transanal opening of the intersphincteric space (TROPIS), fistula-tract laser closure (FiLaC), and fistulectomy.

## Case presentation

**Figure 1:**
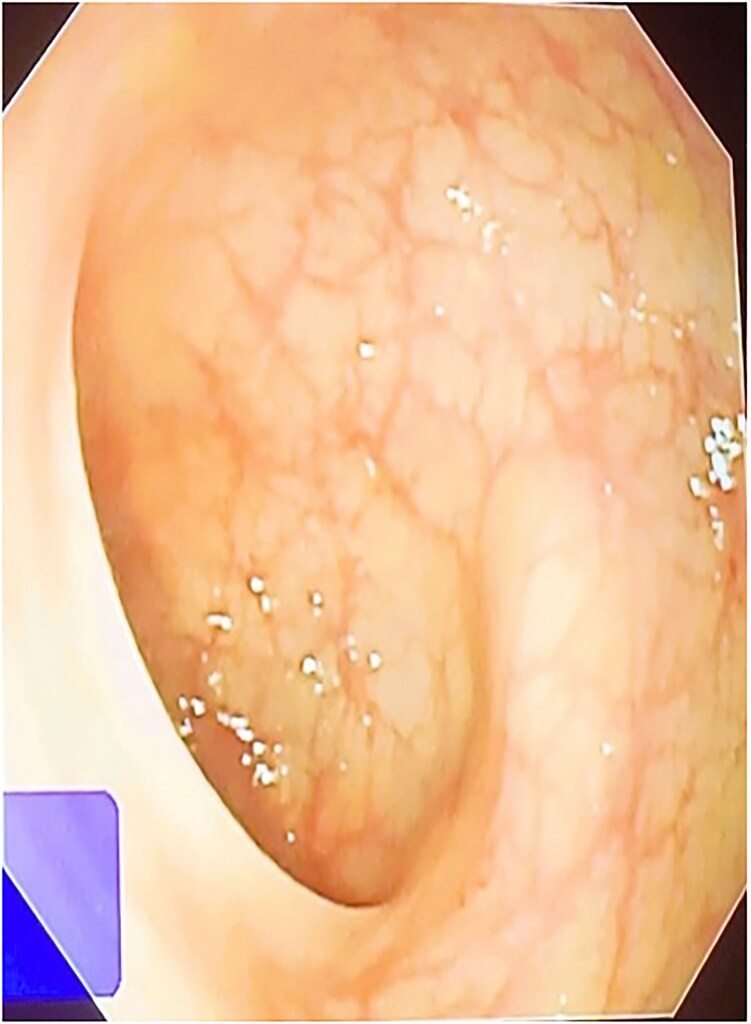
Sigmoidoscopy was found to be normal.

A 42-year-old male patient presented with discharge from anal regions accompanied with pain. Upon rectal examination, an internal opening was observed at 6 o’clock, ~2.5 cm above the anal verge. The external opening was located on the right thigh, 18 cm away, with an indurated tract beneath the skin. Proctoscopy revealed pus discharge from the internal opening. All relevant pre-operative investigations were done (Fig. 1).

**Figure 2:**
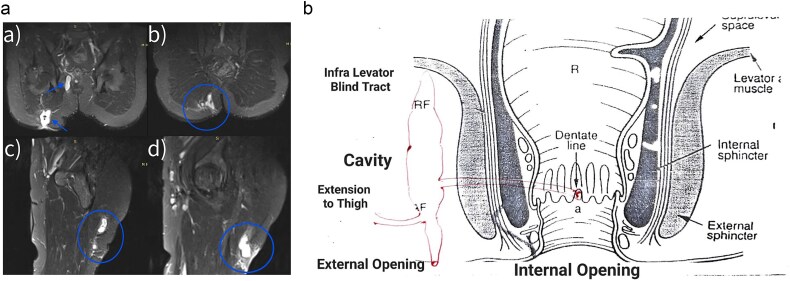
(a) Magnetic resonance imaging of the fistula a trans-sphincteric fistula extending up to the thigh. (b) Diagrammatic representation of fistulous tract.

**Figure 3:**
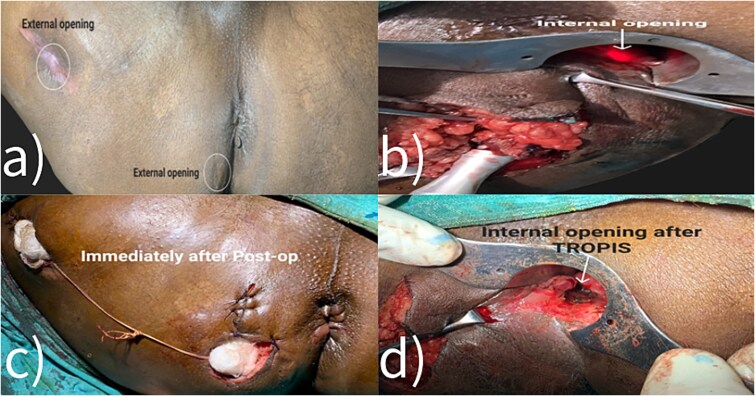
Intraoperative images.

**Figure 4:**
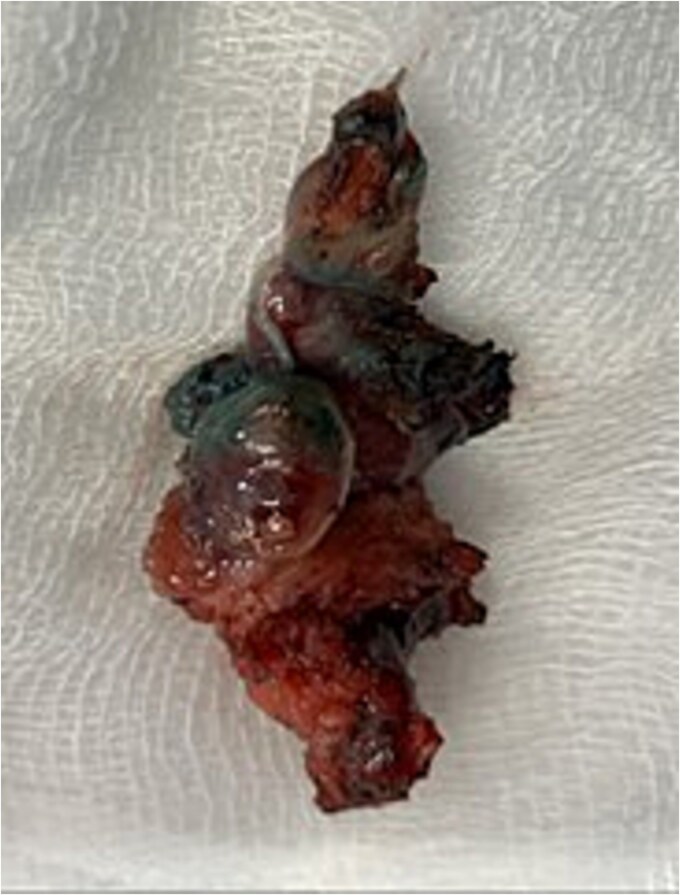
Excised specimen of fistulous tract.

**Figure 5:**
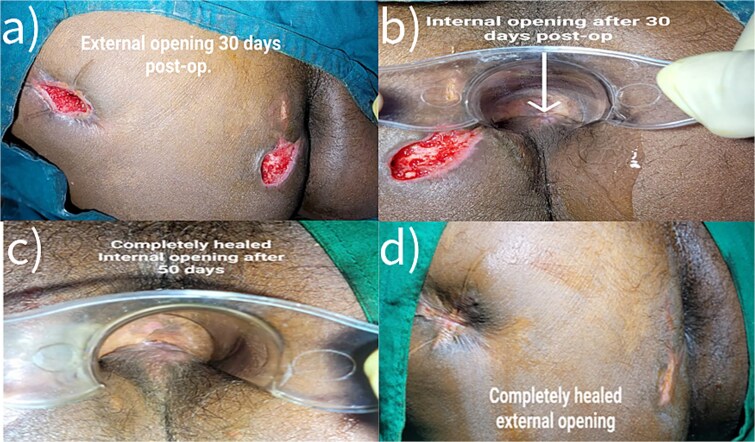
Postoperative Day 30 and Day 50 completely healed.

Fistulectomy was performed in the lithotomy position. The external perianal cutaneous opening in the right buttock was used as the entry point. The tract bifurcated into two branches, forming a Y shape. One branch ended below the right levator ani muscle, while the other branch pierced the external sphincter muscle at the 6 o’clock position. (Figs 2b and 4) Both tracts were removed. The TROPIS procedure was carried out at the internal opening at 6 o’clock. There was a lateral extension of this tract, reaching up to the upper third of the thigh. The distal end of this extension was opened and the tract was thoroughly treated with the radial fiber of the diode laser (Table 1). Due to the length of the fistulous tract, FiLaC was performed (Fig. 3). A draining seton was placed from the thigh to the ischio-rectal fossa. The cavity was packed with roller gauze. The patient was discharged with instructions for regular follow up (Fig. 5).

**Table 1:** Management of fistulous tract that was extended from external opening till mid thigh.

1. End of tracts were excised and wide open.
2. The tract was debrided using scoop.
3. FiLaC done.
4. Middle part of tract ablated using Laser.
5. Tract collapsed.
6. Hence facilatates faster healing.

## Discussion

The treatment of an anal fistula depends on its location and contributing factors. Most fistulas require surgical repair, using different techniques based on the involvement of the internal and external sphincters. Complex fistulas, particularly those caused by Crohn’s disease, may require medical management. We will discuss the most commonly used treatments in detail.

Fistulotomy involves splitting the sphincter muscle and opening the fistula tract. It is highly effective for straightforward fistulas with minimal sphincter involvement, with 90% of appropriately selected patients experiencing recovery [4]. Studies have shown that marsupialization of the fistulotomy edges reduce bleeding and improve post-operative pain management [5].

Endorectal advancement flap surgery is more complex than fistulotomy. It involves closing the internal opening of the fistula tract, removing necrotic tissue, and mobilizing the anorectal mucosa to cover the defect. While the sphincter muscle is not divided during this procedure, there is a risk of compromised sphincter function, with up to 35% of patients reporting incontinence. Success rates vary, ranging from 66% to 87% [4]. Failure is more likely in patients with a history of previous repair attempts, cancer, or Crohn’s disease [6].

Seton drain placement: This two-stage procedure is typically reserved for complicated fistulas. The first stage involves placing a draining seton, which ensures long-term drainage of the fistula tract. Materials such as sutures, vascular loops, or drain devices can be used. The second stage may include a cutting seton, which gradually divides the sphincter complex to decrease the risk of incontinence. In 94% of cases, the fistula can heal completely with this two-step procedure. Fecal incontinence can occur after seton placement, with reported rates of up to 12%.

The ligation of the intersphincteric fistula tract (LIFT) operation is a treatment option for both simple and complex fistulas, with an average success rate of 71%. The procedure involves identifying the internal opening of the fistula and ligating the intersphincteric segment. The infected gland and tract are then removed, and the wound is curetted. Fecal incontinence is rare since the external sphincter is not divided. LIFT can be performed after seton placement as part of a two-stage treatment process [7].

In a fibrin plug and glue, a collagen matrix is used to block or plug the entrance of an internal fistula tract. This therapy is attractive because it does not disrupt the sphincter complex, which helps prevent incontinence. Unfortunately, the success rate of fistula-in-ano treatment is less than 50% [8]. Fibrin glue has also been tested to help speed up the healing of fistula tracts. It has moderate success rates ranging from 14% to 69%, while preserving sphincter function [9]. Initially, both methods were considered unsuccessful, but a more recent study has combined surgical fistula repair with fibrin glue and plugs, which may become a viable treatment option with further investigation [7].

Medical management is required first in patients who are immunocompromised, have systemic symptoms, or have cellulitis near the abscess. HIV patients may benefit from antibiotics and wound cultures [7]. Crohn’s disease patients should also consider medical management. After 54 weeks of treatment, the tumor necrosis factor alpha (TNF-α) monoclonal antibody infliximab showed a fistula closure rate of 36% [10]. If conventional medical therapy is unsuccessful, a staged fistulotomy may be necessary.

Managing complicated anal fistulas is challenging due to their complex anatomy and variable appearance. Complex anal fistulas are typically treated with fistulectomy or fistulotomy with setoning. However, these techniques have high rates of incontinence and recurrence. A meta-analysis shows the recurrence rate following anal fistula can range from 2.5% to 57.1%, depending on the type [10]. Additionally, there is a significant chance that this surgery will result in pain and delayed wound healing [11]. Therefore, managing complex anal fistulas requires an individualized approach that considers the fistula’s structure, presence of additional tracts, and any coexisting conditions.

This complex fistula was cured in 50 days using a diode laser, a vessel sealer, and techniques like fistulectomy, TROPIS, and FiLaC. This approach can effectively cure complex perianal fistulas and may become the recommended approach in the long run.

## Conclusion

Treating complex perianal fistulas with a comprehensive and multidisciplinary strategy is important to reduce the risk of recurrence. The combination of fistulectomy, TROPIS, and FiLaC has shown positive outcomes, giving patients hope for improved health and quality of life. Further research and long-term follow-up studies are needed to verify the long-term efficacy and safety of this approach.
